# Brain Insulin Resistance: Focus on Insulin Receptor-Mitochondria Interactions

**DOI:** 10.3390/life11030262

**Published:** 2021-03-22

**Authors:** Igor Pomytkin, Vsevolod Pinelis

**Affiliations:** 1 Department of Advanced Cell Technologies, I.M. Sechenov First Moscow State Medical University, 119991 Moscow, Russia; ipomytkin@mail.ru; 2 National Medical Research Center for Children’s Health, 119296 Moscow, Russia

**Keywords:** insulin, insulin receptor, brain insulin resistance, mitochondria, brain, neuron, H_2_O_2_, glutamate excitotoxicity

## Abstract

Current hypotheses implicate insulin resistance of the brain as a pathogenic factor in the development of Alzheimer’s disease and other dementias, Parkinson’s disease, type 2 diabetes, obesity, major depression, and traumatic brain injury. A variety of genetic, developmental, and metabolic abnormalities that lead to disturbances in the insulin receptor signal transduction may underlie insulin resistance. Insulin receptor substrate proteins are generally considered to be the node in the insulin signaling system that is critically involved in the development of insulin insensitivity during metabolic stress, hyperinsulinemia, and inflammation. Emerging evidence suggests that lower activation of the insulin receptor (IR) is another common, while less discussed, mechanism of insulin resistance in the brain. This review aims to discuss causes behind the diminished activation of IR in neurons, with a focus on the functional relationship between mitochondria and IR during early insulin signaling and the related roles of oxidative stress, mitochondrial hypometabolism, and glutamate excitotoxicity in the development of IR insensitivity to insulin.

## 1. Introduction

Insulin resistance has long been recognized as a key feature of type 2 diabetes. Historically, the term insulin insensitivity (synonymous with insulin resistance) was used to define the relatively poor glucose response to exogenous insulin exhibited by obese diabetic patients [1]. The meaning of this term has become much broader over time, and insulin resistance is now defined as an impaired biological response to insulin [2] that is not confined just to parameters of glucose metabolism, but includes, in theory, all the biological responses to insulin, e.g., cell growth, differentiation, and protein synthesis. In addition to the classic peripheral insulin-sensitive tissues, such as muscle, liver, and adipose tissue, insulin resistance has been shown to occur in the brain, even in the absence of concurrent type 2 diabetes. Current hypotheses implicate the brain’s insulin resistance as a pathogenic factor in the development of Alzheimer’s disease (AD) and other dementias [3], Parkinson’s disease (PD) [4], type 2 diabetes [5], obesity [6], major depression [7,8], and traumatic brain injury (TBI) [9].

**Figure 1:**
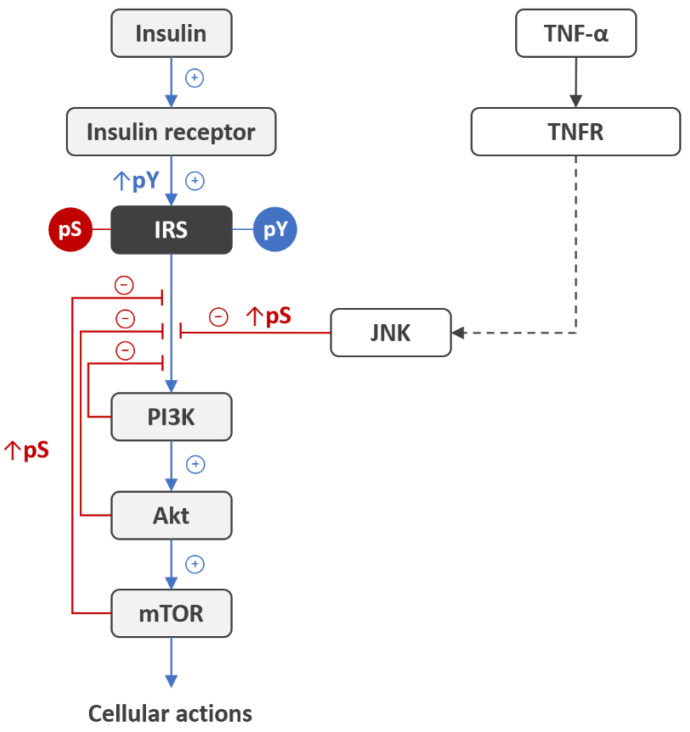
Inhibition of insulin signaling via serine/threonine phosphorylation of IRS proteins. Upon insulin binding to insulin receptor, the receptor tyrosine kinase becomes fully active and evokes tyrosine phosphorylation (pY) of IRS proteins to transduce insulin signal from receptor to downstream effectors PI3K, Akt, and mTOR, which in turn phosphorylate IRS at serine/threonine residues (pS), thereby inhibiting insulin signaling [15,16,17]. This negative feedback autoregulation mechanism is co-opted by hyperinsulinemia, metabolic stress, and inflammation for the development of insulin resistance. In particular, activation of the tumor necrosis factor receptor (TNFR) with tumor necrosis factor-α (TNF-α) leads to activation of downstream c-Jun N-terminal kinase (JNK) and phosphorylation of IRS at the serine residue [27], thereby inducing insulin resistance. Abbreviations: IRS, insulin receptor substrate; pY, phosphotyrosine; pS, phosphoserine; PI3K, phosphatidylinositol 3-kinase; Akt, protein kinase B; mTOR, mammalian target of rapamycin; TNF-α, tumor necrosis factor α; TNFR, tumor necrosis factor receptor; JNK, c-Jun N-terminal kinase.

Insulin elicits its cellular actions by binding to insulin receptors (IRs) presented on the surface of most cells. Evidence suggests that impaired insulin functions in the brain may relate to both insulin deficiency and impaired insulin signal transduction via IRs. The insulin deficiency can occur due to the reduction in insulin transport from the periphery to the brain across the blood-brain barrier [10,11]. It can be compensated by exogenously administered insulin to the brain, e.g., via intranasal route, thereby increasing IRs signaling in AD animal models [12] and improving memory recall in the clinical setting [13]. The impairment of central insulin action can occur also as a result of disturbances in the IR signal transduction, particularly in the activation states of IR and signaling molecules, thereby giving rise to insulin resistance. Insulin signaling is governed by reversible in vivo phosphorylation of the IR itself and downstream effectors, with insulin receptor substrate (IRS) proteins being the first critical node in the signaling cascade, and the nodes further downstream being phosphoinositide 3-kinase (PI3K), protein kinase B (PKB/Akt), and the mammalian target of rapamycin (mTOR) (Figure 1). The IR is a transmembrane protein composed of two extracellular α-subunits (IRα) and two intracellular β-subunits (IRβ), the latter having tyrosine kinase activity. Insulin binding to the IRα evokes fast autophosphorylation of IRβ at Y^1146^, Y^1150^, and Y^1151^ (IR isoform A numbering), upon which the receptor tyrosine kinase becomes fully active [14] and evokes tyrosine phosphorylation of IRS proteins, principally IRS1 and IRS2, to transduce the insulin signal from the IR to downstream effectors PI3K, Akt, and mTOR [5]. Alternative serine/threonine phosphorylation of IRS with downstream kinases PI3K, Akt, and mTOR blocks the insulin signal through the IRS, thereby being a physiological autoregulation mechanism [15,16,17,18,19]. This negative feedback is believed to be co-opted by hyperinsulinemia, metabolic stress, and inflammation for the inhibition of insulin signaling [15,18]. The resulting disbalance between tyrosine and serine/threonine phosphorylation of IRS proteins represents one of the most common mechanisms for development of insulin resistance in the brain [19,20,21,22,23] and peripheral tissues [24,25,26].

A pathologically reduced tyrosine phosphorylation of IRβ, which reflects diminished activity of IR tyrosine kinase, is another common, while much less highlighted, mechanism of insulin resistance [20,28,29]. In particular, it has been reported that insulin-induced tyrosine phosphorylation of IRβ was 26–29% lower in the kinase domain (Y^1146^, Y^1150^, and Y^1151^) and 34–58% lower at the IRS1 docking site (Y^960^) in AD brains compared to age matched controls, even at the same levels of IR and the IR phosphatase PTP1B proteins [20].

This review aims to discuss causes behind the diminished activation of IR in neurons and approaches to treat this kind of insulin resistance.

## 2. Insulin Receptor in the Brain

IRs are widely distributed throughout the brain and are at their highest density in the olfactory bulb, hypothalamus, hippocampus, cerebral cortex, and cerebellum [30,31]. The vast majority of IRs are localized on neurons [32], where they are concentrated at synapses as a component of post-synaptic density (PSD), indicating that the synapse is an important site of specialized insulin signaling in the brain [33].

In contrast to adult peripheral tissues, where long receptor isoform B (IR-B) prevails, neurons almost exclusively express the short isoform A (IR-A), lacking 12 amino acids within the C-terminus of the α-subunit [34,35,36]. The most significant difference between the isoforms is that IR-A binds insulin-like growth factor 2 (IGF2) at physiologically relevant affinity, while IR-B does not [37,38]. In addition, IR-A displays a two-fold higher affinity for insulin than IR-B and shows no negative cooperativity in the insulin binding [39,40,41]. A specific function of IGF2 signaling via IR-A in the brain is the promotion of self-renewal and expansion of neural stem cells [41,42]. Insulin signaling in neurons occurs through two canonic signaling pathways known as the PI3K/Akt and mitogen-activated protein kinase (MAPK) pathways [43,44].

IRs in the brain are involved in the regulation of synaptic plasticity [45]. Insulin facilitates excitatory neurotransmission, mediated by the N-methyl-D-aspartate (NMDA) receptor, by stimulating translocation of functional NMDA receptors to the cell membrane [46] and potentiating NMDA receptor currents in a dose-, time-, and NMDA subunit-specific manner [47,48,49,50,51]. Insulin also facilitates inhibitory neurotransmission through stimulation of the trafficking of the type A γ-aminobutyric acid (GABA_A_) receptor subunits from an intracellular compartment to the membrane surface, thereby increasing the number of functional inhibitory GABA_A_ receptors in the cell membrane [52,53]. The IR is implicated in the modulation of long-term potentiation (LTD) and long-term depression (LTD) [54], learning and memory [55], and regulation of feeding behavior [23]. Although understanding the net functional outcome of insulin on neurotransmission is challenging, the above data suggest a direct link between insulin signaling and synaptic function. In line with this, both synaptic failure and dysfunctional insulin signaling were observed in AD prior to frank neuronal degeneration [20,56,57].

Emerging evidence suggests that insulin signaling also plays a role in glucose metabolism in the brain. The insulin-regulated glucose transporter GLUT4 has been found to be co-expressed with the major neuronal transporter GLUT3 in brain regions related to cognitive behavior, such as the basal forebrain, hippocampus, amygdala, cerebral cortex, and cerebellum [58], and in the hypothalamus that controls food intake and body weight [59]. Insulin stimulates translocation of GLUT4 to the plasma membrane in rat hippocampus [60], increases local glycolytic metabolism, and enhances spatial memory [61]. An inhibition of GLUT4 alone did not impair the spatial memory performance but prevented the insulin’s cognition enhancing effect [62]. Insulin-induced GLUT4 translocation to the neuronal membrane in the hippocampus occurs during periods of high energy demand, such as during learning, suggesting that deregulation of insulin-dependent glucose transport in several brain regions may be a cause of cognitive impairment [63]. For subjects with prediabetes and type 2 diabetes, an association between reduced cerebral glucose metabolic rate and peripheral insulin resistance has been shown even before the onset of mild cognitive impairment [64]. Given such a variety of functions of insulin in the brain, the development of brain insulin resistance can lead to numerous pathological manifestations, especially to those associated with synapse failure and energy metabolism.

## 3. Insulin Receptor Activation: A Role of Redox Priming

It has been long known that autophosphorylation of the trio of tyrosines 1146, 1150, and 1151 (IR-A numbering) within the activation loop (A-loop) of the IRβ kinase domain is critical for stimulation of catalytic activity and biological function of the IR [65,66]. In the unphosphorylated form, IR tyrosine kinase is autoinhibited by interaction of A-loop residues with the active site [67]. However, upon autophosphorylation, the A-loop undergoes a major conformational change, resulting in unrestricted access of IRS proteins and a phosphate donor adenosine triphosphate (ATP) to the active site [68], thereby resulting in a 200-fold increase in the receptor kinase activity [65]. However, it was questionable how insulin binding can induce the autophosphorylation of A-loop tyrosines if both active sites of the IR are locked in the inactive conformation, blocking ATP access to the active site. With these considerations, Schmid et al. suggested that there is a yet unknown intermediate stage in the IR activation process, so called redox priming, where oxidants like hydrogen peroxide (H_2_O_2_) facilitate, while antioxidants inhibit, the insulin-induced IRβ autophosphorylation [69]. An oxidative modification of cysteine residues within IRβ was proposed as the structural basis of the “redox priming”, with Cys^1138^ in the proximity of catalytic aspartate 1132 being the most prominent candidate for the priming, since the non-oxidizable IR mutant Cys^1138Ala^ was the only IR cysteine mutant that showed defective kinase activity in functional experiments [70]. The idea of redox priming was supported by the fact that insulin stimulation itself leads to generation of endogenous H_2_O_2_ in fat cells [71,72]. Therefore, the insulin-induced H_2_O_2_ could be the priming factor facilitating IR autophosphorylation in vivo. It was later found that the role of H_2_O_2_ is not restricted by the redox priming of IR and also includes inhibition of protein tyrosine phosphatase PTP1B, which inactivates the IR by dephosphorylating A-loop phosphotyrosines [73]. Collectively, the insulin-induced H_2_O_2_ plays a role of net positive regulator of IR activation through its concerted actions on the opposite activities of IR kinase and PTP1B phosphatase.

Evidence from experiments in transgenic animals supported the significance of endogenous H_2_O_2_ generation for proper insulin actions. Glutathione peroxidase (Gpx1) is a highly effective scavenger for H_2_O_2_, with a second-order rate constant of about 10^7^ M^−1^ s^−1^ [74]. It has been reported that mice overexpressing Gpx1 were hyperinsulinemic, insulin resistant, and obese and had a 70% reduction in the insulin-stimulated tyrosine phosphorylation of IRβ compared to the wild-type control [75]. On the contrary, mice lacking Gpx1 were protected from insulin resistance induced by a high-fat diet, while administration of N-acetylcysteine (NAC), the Gpx1 substrate [76], rendered them insulin-resistant and increased fasting glucose levels [77].

At least two insulin-sensitive sources of H_2_O_2_ have been found in peripheral tissues, nicotinamide adenine dinucleotide phosphate (NADPH) oxidase 4 (NOX4) in fat cells [78,79,80] and mitochondria in liver and heart preparations [81,82]. A transient insulin-induced NADPH-dependent generation of reactive oxygen species (ROS) has been reported in the hypothalamus, with peak ROS release not earlier than 15 min post-stimulation [83], with these ROS being involved in the regulation of energy metabolism and food intake [83,84].

## 4. Critical Role of Redox Signaling in the Activation of the Neuronal Insulin Receptor

In 2007, we showed for the first time that insulin stimulation generates a spike of H_2_O_2_ in neurons and that NAC, the Gpx1-dependent H_2_O_2_ scavenger, completely abrogated both the insulin-induced H_2_O_2_ and autophosphorylation of IRβ at Y1150/1151, thereby suggesting that the H_2_O_2_ signal is a critical requirement for the activation of the IR in the neurons [85]. Further kinetic studies have demonstrated that the insulin-induced H_2_O_2_ signal has a duration of about 15–30 s and a peak at 5 s post-stimulation [86]. Comparison of timings of the insulin-induced H_2_O_2_ signal and IR autophosphorylation has demonstrated that they peaked at 5 s and at 10 min, respectively, indicating that the H_2_O_2_ signal precedes the activation of the IR in the neurons [86]. The insulin-induced autophosphorylation of the IR was extremely ultrasensitive to H_2_O_2_ scavenging (a sigmoidal dose-response with Hill’s slope of about 8), indicating the presence of a certain threshold level of the H_2_O_2_ signal, below which IR autophosphorylation does not occur, even in the presence of the highest insulin concentrations [86]. Conversely, IR autophosphorylation occurs only when the H_2_O_2_ signal has surpassed the threshold. The insulin dose-response on IR autophosphorylation in neurons was found to be gradual, with a Hill’s slope of about 1 [86]. So, the activation of the IR in the neurons depends on two variables, gradually on the concentration of extracellular insulin and stepwise on the magnitude of intracellular insulin-induced H_2_O_2_ signal. The latter type of dependence is known as “all-or-nothing”, i.e., the IR activation performing either completely or not at all, depending on whether the H_2_O_2_ signal can or cannot exceed a certain threshold.

In view of the high significance of H_2_O_2_ signal for the activation of the IR in the neurons, a pathological increase in the activity of antioxidant enzyme scavenging H_2_O_2_ in cells may be the factor contributing to insulin resistance. A marked increase of expression of antioxidant enzymes in a region- and cell type-specific manner has been shown in the brains of patients with AD and other neurodegenerative disorders, presumably as a compensatory defense response to oxidative stress [87,88]. Levels of mRNA for Gpx, catalase (CAT), and glutathione reductase (GSSG-R) were elevated in the hippocampus of AD patients [89]. Protein levels of peroxiredoxins PRDX1 and PRDX2 were significantly increased in the brains of AD and Down Syndrome (DS) patients [90]. PRDX2 was significantly increased in the frontal cortex of DS, AD, and PD patients [91], the hippocampus of AD patients [92], and the striatum of Huntington’s disease (HD) patients [93]. Among others, the overexpressed Gpx [89] and peroxiredoxins PRDX1, PRDX2, and PRDX4 [94], the most fast and effective antioxidant systems for H_2_O_2_ elimination, may represent a barrier for the insulin-induced H_2_O_2_ signal, thereby contributing to reduced activation of the IR in the neurons in response to insulin.

## 5. G Protein Activity in the Activation of the Neuronal Insulin Receptor

The inhibitory G protein activity is involved in the generation of the insulin-induced H_2_O_2_ signal in neurons [86]. Both the insulin-induced H_2_O_2_ signal and IR phosphorylation were completely abrogated by pertussis toxin (PTX), a classic inhibitor of Gi/o protein-receptor coupling, during insulin stimulation [86]. It has long been known that the Gαi2 isoform is the specific G protein that is recruited by IRs in peripheral tissues and affects IR autophosphorylation proportionally to the extent of such an association [95]. Mice expressing constitutively active Gαi2 had enhanced insulin signaling to GLUT4 [96,97] and markedly amplified tyrosine phosphorylation of the IR in fat and skeletal muscle in vivo [98]; on the contrary, mice deficient in Gαi2 expression had reduced insulin sensitivity in peripheral tissues [99]. However, much less is known about IR and Gαi2 relationships in the brain. No significant differences were found between Gαi2 levels in the brains of young and aged controls and patients with AD [20,100,101], indicating no role for Gαi2 in the development of brain insulin resistance in AD.

## 6. Mitochondrial Signaling Is an Integral Part of the Insulin Receptor Activation Process in Neurons

We found that the insulin-induced H_2_O_2_ signal in neurons was inhibited with malonate, an inhibitor of mitochondrial complex II at the flavin site (II_F_), indicating the involvement of mitochondria in the generation of the insulin-induced H_2_O_2_ signal [85,86]. In full agreement with this, succinate enhanced and malonate dose-dependently inhibited, completely inhibiting at the highest concentrations, the insulin-induced autophosphorylation of IRβ (i.e., the receptor activation) in neurons [85,86,102]. The malonate dose-response on IR autophosphorylation was sigmoidal, with a Hill’s slope of more than 3. So, the activation of IR is ultrasensitive to the activity of mitochondrial complex II, and even a small change in the rate of succinate oxidation at the II_F_ site around a certain threshold can have a dramatic effect on the IR autophosphorylation [86]. This suggests that mitochondrial complex II is a critical regulatory point in the activation of the neuronal IR, with the activation occurring either completely or not at all, depending on whether the rate of succinate oxidation at the flavin site II_F_ can or cannot exceed a certain threshold.

At malonate concentrations that completely inhibited the IR autophosphorylation, the insulin-induced H_2_O_2_ signal was also completely abolished, suggesting that succinate oxidation at complex II is the only source of the insulin-induced H_2_O_2_ in neurons. The reverse electron transport from complex II to complex I, a previously reported major mechanism of H_2_O_2_ generation in brain mitochondria respiring on supra-physiological millimolar succinate concentrations [103,104,105], appears to have no important role in the production of the insulin-induced H_2_O_2_, since rotenone, an inhibitor of complex I, hardly influenced this process as well as on IR autophosphorylation during insulin stimulation [86]. Quinlan et al. have shown recently that mitochondrial complex II itself can generate H_2_O_2_ at high rates in the presence of physiological micromolar succinate concentrations, with a malonate-sensitive flavin site II_F_ within complex II being the source of the H_2_O_2_ [106]. In this context, the site II_F_ within the mitochondrial complex II appears to be the direct source of the insulin-induced H_2_O_2_ signal, with H_2_O_2_ being producing by succinate oxidation with molecular oxygen by the reaction: succinate + O_2_ → fumarate + H_2_O_2_.

It should be noted that the insulin effect on mitochondrial complex II activity has long been known. Experiments with ^14^C-labeled succinate have demonstrated that an increase in succinate oxidation at complex II occurs almost immediately upon stimulation of cells with insulin, representing one of the fastest metabolic effects of insulin [107,108]. In line with this, the pre-treatment of liver and heart preparations with insulin markedly increased rates of H_2_O_2_ production in mitochondria respiring at micromolar succinate concentrations [91]. The findings that mitochondrial complex II is involved in the insulin-induced H_2_O_2_ signaling and IR autophosphorylation extends this picture by showing that the relationship between IR and complex II is bidirectional and has a control function in IR activation.

**Figure 2:**
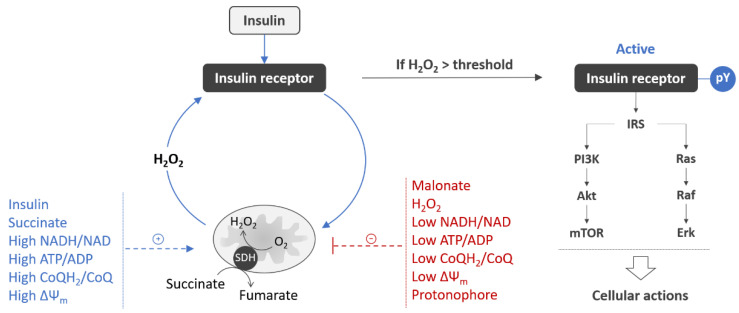
Mitochondrial controls for activation of insulin receptor in neurons. Insulin stimulation evokes a mitochondrial H_2_O_2_ signal, which generation requires succinate oxidation at SDH [85,86]. IR autophosphorylation (pY) occurs only if the H_2_O_2_ signal exceeds a certain threshold [85,86]. Upon the autophosphorylation, IR becomes fully active and elicits its cellular actions through signaling via two canonical PI3K/Akt/mTOR and Ras/Raf/Erk pathways [43,44]. Given that generation of the insulin-induced H_2_O_2_ signal requires high SDH activity [85,86], factors affecting this activity may play a role of positive (in blue) or negative (in red) regulators of IR activation. SDH activity is enhanced by insulin [81,107,108], succinate [109], high ΔΨ_m_ [110], and high NADH/NAD, ATP/ADP, and CoQH_2_/CoQ ratios [111,112]. SDH activity is downregulated by malonate, H_2_O_2_ [113,114], mitochondrial depolarization [110], and low NADH/NAD, ATP/ADP, and CoQH_2_/CoQ ratios [111,112], thereby predisposing to the development of insulin resistance. Abbreviations: H_2_O_2_, hydrogen peroxide; SDH, succinate dehydrogenase; IR, insulin receptor; PI3K, phosphatidylinositol 3-kinase; Akt, protein kinase B; mTOR, mammalian target of rapamycin; Ras, rat sarcoma small GTPase; Raf, rapidly accelerated fibrosarcoma kinase; Erk, extracellular signal-regulated kinase; ΔΨm, mitochondrial inner membrane potential; NADH/NAD, reduced-to-oxidized nicotinamide adenine dinucleotide ratio; ATP/ADP, adenosine triphosphate-to-adenosine diphosphate ratio; CoQH_2_/CoQ, reduced-to-oxidized coenzyme Q ratio.

In line with the above background, in theory, any disturbance in the generation of the insulin-induced H_2_O_2_ signal may result in less tyrosine phosphorylation of IRβ, with low activity of mitochondrial complex II during insulin stimulation being one of the prominent causes for insulin resistance in the brain (Figure 2).

Mitochondrial complex II, also known as succinate-ubiquinone oxidoreductase or succinate dehydrogenase (SDH), oxidizes succinate to fumarate in the tricarboxylic acid cycle (TCA) and reduces coenzyme Q (CoQ) in the respiratory chain. During succinate oxidation, two electrons are transferred from succinate to the flavin at site II_F_ and then to CoQ at site II_Q_, to supply the respiratory chain with reducing equivalents. SDH exists in two forms, either in the active form stabilized by binding with succinate or in the non-active form stabilized as a 1:1 complex with oxaloacetate [115]. As the oxaloacetate binding affinity to the reduced form of the enzyme is at least one order of magnitude less than that to the oxidized form, SDH represents a redox-regulated switch, activated upon reduction, when it liberates oxaloacetate, and inhibited upon oxidation [116]. Physiological activators of SDH are succinate [109] and the reduced form of coenzyme Q (CoQH_2_) [112]. SDH activity depends on the electron flux from complex I [111], since CoQH_2_ is largely produced at complex I by the reduction of CoQ with reduced nicotinamide dinucleotide (NADH). The rapid deactivation of SDH occurs during extensive oxidation of CoQH_2_, occurring at low ATP/ADP ratio, e.g., in the presence of protonophores inducing mitochondrial depolarization [110]. H_2_O_2_ exposure also decreases SDH activity through the enhancement of oxaloacetate binding [113,114]. The last fact indicates an exact molecular link between oxidative stress and low SDH activity, which may lead to less activation of the IR during insulin stimulation. In summary, mitochondrial hypometabolism and oxidative stress are the factors that reduce SDH activity, thereby predisposing to less activation of the IR during insulin stimulation and resulting in the development of insulin resistance.

Mitochondrial depolarization has been found to be one other cause for low activity of the IR in neurons during insulin stimulation [85,86]. The mitochondrial inner membrane potential (ΔΨm) is an essential component in the process of energy storage during oxidative phosphorylation. It is generated by proton transfers at complexes I, III and IV and, together with the proton gradient, forms the transmembrane potential of hydrogen ions which is used to make ATP. Protonophore carbonyl cyanide p-trifluoro-methoxyphenyl hydrazone (FCCP)-induced inhibition of the insulin-induced IR autophosphorylation occurs in parallel with a decrease in the ΔΨ_m_. At highest FCCP concentration, resulting in ΔΨ_m_ collapse, both the insulin-induced IR autophosphorylation and H_2_O_2_ signal were completely abrogated.

The signs of mitochondrial dysfunction, such as reduced ATP levels and decreased ΔΨ_m_, have been demonstrated in AD [117,118] and TBI [119], for which brain insulin resistance is a concomitant condition.

It still remains to be explored whether the mitochondrial control of IR activation is a neuron-specific mechanism. It should be noted that neuronal IR are localized predominantly in the PSD of dendritic spines, which are poor in mitochondria at rest, but become enriched with mitochondria during repetitive depolarization due to activity-regulated mitochondrial fusion/fission and mitochondria trafficking [120]. In this context, the mitochondrial H_2_O_2_ signaling in neurons may be a control mechanism for the selective activation of IRs only in active synapses.

## 7. Glutamate Excitotoxicity Impairs Activation of the Neuronal Insulin Receptor

Glutamate excitotoxicity is a common pathological condition that affects mitochondrial metabolism and complex II activity in the brain, thereby being a prominent candidate for the role of inducer of brain insulin resistance. Glutamate is the major excitatory neurotransmitter that is involved in most normal brain function, such as cognition, memory, and learning, through binding to several types of glutamate receptors [121]. However, an excessive glutamate release to the synaptic cleft may induce a specific pathophysiological process called excitotoxicity. The glutamate-induced activation of the ionotropic NMDA receptors, followed by a Ca^2+^ influx into the cell, is generally considered to be central to the development of excitotoxicity [122,123,124]. The Ca^2+^ influx is biphasic and an initial rapid increase in the intracellular free Ca^2+^ concentration ([Ca^2+^]_i_) is followed by a larger secondary [Ca^2+^]_i_ increase, along with a marked decrease in ΔΨ_m_, SDH activity, and ATP production [125,126,127,128,129]. The irreversible secondary [Ca^2+^]_i_ increase, known as delayed calcium deregulation, is postulated to be a point-of-no-return in excitotoxicity, i.e., events occurring downstream of this point are considered to influence the timing of cell death without altering its inevitability [130].

**Figure 3:**
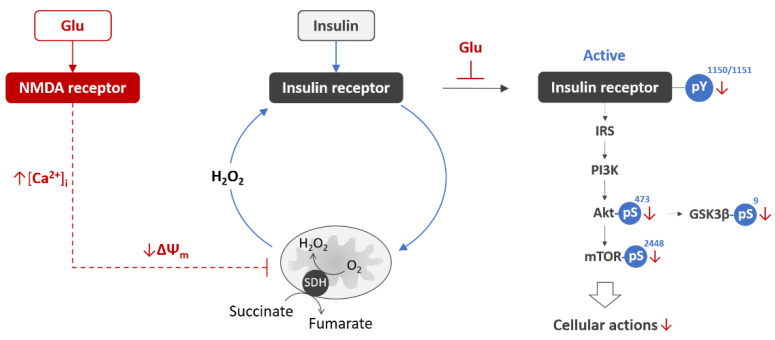
Glutamate excitotoxicity induces acute neuronal insulin resistance. Glutamate (Glu) binding to NMDA receptor evokes rapid increase in the intracellular free Ca^2+^ concentration ([Ca^2+^]_i_), followed by decrease in mitochondrial ΔΨ_m_ [127,131]. Within minutes, when the glutamate-induced mitochondrial depolarization occurred, insulin evoked less tyrosine phosphorylation of IR Y^1150/1151^, and less serine phosphorylation of Akt S^473^, mTOR S^2448^, and GSK3β S^9^ [29], indicating the development of acute neuronal insulin resistance as an early pathological event associated with excitotoxicity. Abbreviations: Glu, glutamate; NMDA, N-methyl-D-aspartate; [Ca^2+^]i, intracellular calcium concentration; ΔΨm, mitochondrial inner membrane potential; H_2_O_2_, hydrogen peroxide; SDH, succinate dehydrogenase; IRS, insulin receptor substrate; PI3K, phosphatidylinositol 3-kinase; Akt, protein kinase B; mTOR, mammalian target of rapamycin; GSK3β, glycogen synthase kinase 3 β; pY, phosphotyrosine; pS, phosphoserine.

Emerging evidence suggests that there is a functional relationship between IR and NMDA receptors in health and disease. Both types of receptors are co-localized in the PSD of the synapses [33]. The IR is involved in the regulation of NMDA receptor trafficking [46] and the potentiation of NMDA receptor currents in a dose-, time-, and NMDA subunit-specific manner [47,48,49,50,51]. The NMDA receptor is involved in the inhibition of tyrosine phosphorylation of the IR in cortical and hippocampal cultures of neurons with soluble β-amyloid oligomers [29]. The amyloid-like effect was achieved with glutamate added one hour after the insulin stimulation, i.e., at times when the active IR undergoes dephosphorylation and deactivation [29]. Glutamate also affects the activation of the IR and downstream effectors, when being added prior to insulin exposure, thereby developing acute neuronal insulin resistance within minutes (Figure 3) [131].

At times where significant mitochondrial depolarization has been achieved due to glutamate-evoked massive influxes of Ca^2+^ into the cells, insulin induced 48% less activation of the IR kinase domain (assessed by IR tyrosine phosphorylation, pY^1150/1151^), 72% less activation of Akt (assessed by Akt serine phosphorylation, pS^473^), 44% less activation of mTOR (assessed by mTOR pS^2448^), and 38% less inhibition of glycogen synthase kinase β (GSK3β) (assessed by GSK3β pS^9^) compared with respective controls [131]. Thus, the glutamate-induced development of acute neuronal insulin resistance represents one of the earliest pathological events in excitotoxicity, which occurs at the level of activation of the IR in the neurons.

It has already been shown that glutamate excitotoxicity is implicated in the pathogenesis of TBI [122] and AD [132]. However, the existence of the causal relationship between excitotoxicity and brain insulin resistance indicates that list of disorders associated with brain insulin resistance is much broader and may include stroke [133], PD [134], HD, amyotrophic lateral sclerosis [135], depression, autism spectrum disorder, schizophrenia [136], and multiple sclerosis [137], for which glutamate excitotoxicity has already been demonstrated as a pathogenic factor.

The relationship between IR activation and glutamate excitotoxicity appears to be bidirectional, since insulin itself activates mitochondrial metabolism. Although hyperinsulinemia and a long-term insulin exposure have been shown to exacerbate glutamate excitotoxicity through inducing insulin resistance [138], in contrast, a short-term insulin treatment protects neurons against glutamate excitotoxicity [128]. The short-term stimulation of cortical neurons with insulin prior to glutamate exposure protects them from the NMDA receptor-mediated increase in [Ca^2+^], thereby preventing the mitochondrial depolarization, decrease in ATP levels, and decrease in oxygen consumption rates due to the preservation of spare respiratory capacity (SRC) [120]. SRC, also known as the reserve respiratory capacity, refers to the measure of the amount of extra ATP that can be produced by oxidative phosphorylation in case of an increase in energy demand. It has been shown that mitochondrial complex II is a source of SRC [139]. Given that insulin enhances succinate oxidation at complex II [107,108], the insulin protective action against glutamate excitotoxicity seems to relate to the insulin-induced improvement of complex II-dependent ATP production and mitochondrial metabolism.

It should be noted that the discussed above functional relationship between IR activation and glutamate excitotoxicity is part of more complex relationships between deficient insulin signaling and Ca^2+^ dyshomeostasis in neurons that are associated with brain aging [140,141]. Insulin and insulin sensitizers have been shown to target several hippocampal Ca^2+^-related processes affected by aging, including larger Ca^2+^ transients and Ca^2+^-dependent afterhyperpolarizations [140], with the reduction of voltage-gated calcium currents being implicated in the mechanisms of these insulin effects [142].

## 8. Conclusions and Perspectives

Brain insulin resistance leads to a variety of abnormalities, both related and unrelated to brain glucose utilization, with deterioration of cognitive function and energy metabolism being the most recognized. The disbalance between tyrosine and serine/threonine phosphorylation of IRS protein is the most common cause of insulin resistance associated with metabolic stress, hyperinsulinemia, and inflammation. The diminished autophosphorylation (i.e., activation) of the IR during insulin stimulation is another reported cause of brain insulin resistance.

In this review, we summarized the data on the functional relationship between activation of the IR in the neurons and mitochondrial redox signaling during insulin stimulation. The insulin-induced mitochondrial H_2_O_2_ signal occurring from complex II is an integral part of the IR autophosphorylation process in neurons, with the IR activation occurring either completely or not at all, depending on whether the H_2_O_2_ signal can or cannot exceed a certain threshold. It remains unexplored whether the mitochondrial control of IR activation is a neuron-specific mechanism or a more general phenomenon. Neuronal IRs are localized predominantly in the PSD of dendritic spines, which are poor in mitochondria at rest, but become enriched with mitochondria in periods of synaptic activity. In this context, the mitochondrial H_2_O_2_ signaling in neurons may be a control mechanism for the selective activation of the IR only in the active synapses.

Given the critical role of H_2_O_2_ signaling in IR activation, factors downregulating the mitochondrial H_2_O_2_ signal may lead to less activation of the IRs and the development of brain insulin resistance. The incomplete list of such factors includes oxidative stress, glutamate excitotoxicity, the overexpression of antioxidant enzymes compensatory to oxidative stress, mitochondrial depolarization, and mitochondrial hypometabolism manifested as low ATP/ADP, NADH/NAD, and CoQH_2_/CoQ ratios.

In this context, the interventions aimed at improving mitochondrial metabolism represent a reasonable approach to the treatment of brain insulin resistance at the level of IR activation through the improvement of insulin-induced H_2_O_2_ signaling in neurons.

## Data Availability

Not applicable.

## Abbreviations

AD, Alzheimer’s disease; Akt, protein kinase B; CAT, catalase; GABAA, type A γ-aminobutyric acid receptor; GLUT3, glucose transporter 3; GLUT4, glucose transporter 4; Gpx1, glutathione peroxidase; GSK3β, glycogen synthase kinase 3 β; GSSG-R, glutathione reductase; IR, insulin receptor; IRα, insulin receptor α-subunit; IRβ, insulin receptor β-subunit; IR-A, insulin receptor A isoform; IR-B, insulin receptor B isoform; IRS, insulin receptor substrate; IGF2, insulin-like growth factor 2; H_2_O_2_, hydrogen peroxide; JNK, c-Jun N-terminal kinase; PSD, post-synaptic density; PI3K, phosphoinositide 3-kinase; mTOR, mammalian target of rapamycin; pY, phosphotyrosine; pS, phosphoserine; TNF-α, tumor necrosis factor α; TNFR, tumor necrosis factor receptor; MAPK, mitogen-activated protein kinase; NMDA, N-methyl-D-aspartate; LTD, long-term potentiation; LTD, long-term depression; NAC, N-acetylcysteine; NOX4, nicotinamide adenine dinucleotide phosphate oxidase 4; ROS, reactive oxygen species; PD, Parkinson’s disease; PRDX1, peroxiredoxin 1; PRDX2, peroxiredoxin 2; PRDX4, peroxiredoxin 4; PTX, pertussis toxin; Ras, rat sarcoma small GTPase; Raf, rapidly accelerated fibrosarcoma kinase; Erk, extracellular signal-regulated kinase; ΔΨm, mitochondrial inner membrane potential; NADH/NAD, reduced-to-oxidized nicotinamide adenine dinucleotide ratio; ATP/ADP, adenosine triphosphate-to-adenosine diphosphate ratio; CoQH_2_/CoQ, reduced-to-oxidized coenzyme Q ratio; SRC, spare respiratory capacity.
